# Epidemiological and clinical characteristics of the first 500 confirmed COVID-19 inpatients in a tertiary infectious disease referral hospital in Manila, Philippines

**DOI:** 10.1186/s41182-021-00340-0

**Published:** 2021-06-12

**Authors:** Kristal An Agrupis, Chris Smith, Shuichi Suzuki, Annavi Marie Villanueva, Koya Ariyoshi, Rontgene Solante, Elizabeth Freda Telan, Kelly Anne Estrada, Ann Celestyn Uichanco, Jocelyn Sagurit, Joy Calayo, Dorcas Umipig, Zita dela Merced, Fe Villarama, Efren Dimaano, Jose Benito Villarama, Edmundo Lopez, Ana Ria Sayo

**Affiliations:** 1 School of Tropical Medicine and Global Health, Nagasaki University, Nagasaki, Japan; 2 San Lazaro Hospital–Nagasaki University Collaborative Research Office, Manila, Philippines; 3 Department of Clinical Research, London School of Hygiene and Tropical Medicine, London, UK; 4 San Lazaro Hospital, Manila, Philippines; 5 Institute of Tropical Medicine, Nagasaki University, Nagasaki, Japan

**Keywords:** COVID-19, Philippines, Epidemiology, Low-resource setting, Healthcare workers, Mortality

## Abstract

**Background:**

The Philippines has been one of the most affected COVID-19 countries in the Western Pacific region, but there are limited data on COVID-19-related mortality and associated factors from this setting. We aimed to describe the epidemiological and clinical characteristics and associations with mortality among COVID-19-confirmed individuals admitted to an infectious diseases referral hospital in Metro Manila.

**Main text:**

This was a single-centre retrospective analysis including the first 500 laboratory-confirmed COVID-19 individuals admitted to San Lazaro Hospital, Metro Manila, Philippines, from January to October 2020. We extracted clinical data and examined epidemiological and clinical characteristics and factors associated with in-hospital mortality. Of the 500 individuals, 133 (26.6%) were healthcare workers (HCW) and 367 (73.4%) were non-HCW, with HCW more likely presenting with milder symptoms. Non-HCW admissions were more likely to have at least one underlying disease (51.6% vs. 40.0%; *p* = 0.002), with hypertension (35.4%), diabetes (17.4%), and tuberculosis (8.2%) being the most common. Sixty-one (12.2%) died, comprising 1 HCW and 60 non-HCW (0.7% vs. 16.3%; *p* < 0.001). Among the non-HCW, no death occurred for the 0–10 years age group, but deaths were recorded across all other age groups. Compared to those who recovered, individuals who died were more likely to be older (*p* < 0.001), male (*p* = 0.015), report difficulty of breathing (*p* < 0.001), be HIV positive (*p* = 0.008), be intubated (*p* < 0.001), categorised as severe or critical (*p* < 0.001), have a shorter mean hospital stay (*p* < 0.001), or have an additional diagnosis of pneumonia (*p* < 0.001) or ARDS (*p* < 0.001).

**Conclusion:**

Our analysis reflected significant differences in characteristics, symptomatology, and outcomes between healthcare and non-healthcare workers. Despite the unique mix of cohorts, our results support the country’s national guideline on COVID-19 vaccination which prioritises healthcare workers, the elderly, and people with comorbidities and immunodeficiency states.

## Background

The COVID-19 pandemic caused by the severe acute respiratory syndrome coronavirus-2 (SARS-CoV-2) is now in its second year [1, 2]. The Philippines, already with a high burden of infectious disease [3], has been one of the hardest hit countries in the Western Pacific region [4]. As of April 2021, the densely populated National Capital Region (NCR) has been the epicentre of COVID-19, contributing to almost half the cases in the Philippines [5, 6]. Significant progress has been made in a short period of time in terms of understanding the virus’ pathogenesis, transmission, and symptomatology [7, 8]. Therapeutic modalities have been evaluated and vaccines developed [9–11]. While initial doses of the COVID-19 vaccine have been given to priority populations (healthcare workers, the elderly, and those with comorbidities) in the Philippines since March 2021 [9, 10], only 739,000 individuals had received at least one dose of vaccine, comprising < 1% of the total population [9]. Hospitals in the NCR are still experiencing a heavy influx of patients presenting with severe to critical symptoms of COVID-19. One way to help alleviate the burden in the hospitals is to further identify those who would benefit most from the available COVID-19 vaccines. While numerous publications are available for Western and high-income settings, to date, there are still limited and underrepresented data among Filipinos and Asians in low-resource settings.

We previously reported an analysis of the first 100 individuals with suspected COVID-19 admitted to San Lazaro Hospital (SLH), a tertiary infectious diseases hospital in Metro Manila, during the first months of the pandemic [11]. Being the national infectious disease referral centre in the country, SLH caters mostly to patients with communicable diseases. When the pandemic started, adjustments in the admission policy were made to provide care to COVID-19 patients, particularly prioritising healthcare workers serving as frontliners in the fight against the disease. The policy for admission has changed over time, with subsequent revisions in the criteria for admission (i.e. cases with mild symptoms are isolated at home or in an isolation facility and not admitted to a hospital) in order to prioritise those with more severe presentations. In this follow-up paper, we aim to describe the epidemiological and clinical characteristics and associations with mortality among the first 500 laboratory-confirmed COVID-19 inpatients from the same hospital, with a view to identifying individuals most at risk who could be prioritised for vaccination.

## Main text

We conducted a secondary analysis of the first 500 laboratory-confirmed COVID-19 inpatients at SLH, from January 25, 2020, to October 24, 2020. Anonymised data on confirmed and suspected cases within the hospital from COVID-19 case investigation forms (CIF) were provided by the SLH Epidemiology Department (SLH-ED). We limited our analysis to individuals with complete data on case classification and patient outcome. Clinical status (asymptomatic, mild, moderate, severe) was assessed according to the Philippine Department of Health’s Interim Guidelines on the COVID-19 Disease Severity Classification and Management [12]. Available laboratory data on cycle threshold (Ct) value was collected from the hospital’s laboratory department. The Philippines’ Department of Health (DOH) defines a “confirmed case” of COVID-19 as “any individual, irrespective of the presence or absence of clinical signs and symptoms, who is laboratory-confirmed for COVID-19 in a test conducted at the national or subnational reference laboratory, and/or officially accredited laboratory testing facility”. For the purpose of this analysis, we included individuals categorised in the CIF as confirmed cases of COVID-19. We clarified any unclear information in the dataset with the staff of the SLH-ED. We analysed the descriptive statistics of cases, deaths, and recoveries by socio-demographics and clinical presentation. We used proportions and percentages to describe the characteristics of the study population. Continuous data were described as means (standard deviation (SD)) if the data was normally distributed; otherwise, medians (interquartile range) were used. We calculated time in days from the onset of symptoms to hospital admission, and length of hospitalisation until death or discharge. Categorical variables were analysed using χ^2^ testing. Analysis of associations with mortality was restricted to non-healthcare workers, given that all the deaths apart from one occurred in this group. Stata IC 16.1 was used for all analyses. The study was approved by the SLH research ethics and review unit (Ref: SLH-RERU-2020-022-I) and the School of Tropical Medicine and Global Health, Nagasaki University Ethical Committee (NU_TMGH_2020_119_1).

Table 1 presents the epidemiological and clinical characteristics of the 500 individuals included in the analysis, comparing 133 (26.6%) HCW and 367 (73.4%) non-HCW. Most were aged over 20 with a median age of 48 years (IQR 34–61). HCW tended to be younger (*p* < 0.001). All 16 individuals aged under 20 were non-HCW. Just over half were males (55.8%), similar for both groups. All (99.6%) were Filipino nationals apart from two individuals from China. The majority (73.0%) could not identify a possible exposure to COVID-19; however, HCW were more likely to report risk of exposure in the workplace (*p* < 0.001). Non-HCW were more likely to have at least one underlying disease (55.9% vs. 40.0%; *p* = 0.002), with hypertension (38.1%), diabetes (20.2%), and tuberculosis (10.1%) being the most common. The predominant symptoms among the admitted cases were cough (76.2%), fever (56.2%), and difficulty of breathing (37.0%). Non-HCW were more likely to have symptoms of fever (*p* < 0.001), cough (*p* < 0.001), difficulty of breathing (p < 0.001), malaise or fatigue (*p* = 0.034), and loss of appetite (*p* < 0.002), whereas HCW were more likely to report coryzal symptoms (*p* = 0.003). All admitted individuals had symptoms. HCW were more likely to be classified as mild or moderate whereas non-HCW were more likely to be severe or critical cases (*p* < 0.001). Twenty-nine (5.8%) individuals needed mechanical ventilator support, all non-HCW. The median duration between the onset of symptoms to admission was 6 days (IQR 4–9 days), less for HCW compared with non-HCW (5.5 days vs. 7 days; *p* < 0.001). The median duration of hospital stay was 10 days (IQR 7–14 days) with no difference between HCW and non-HCW. HCW were more likely to be admitted within 14 days from the symptom onset than non-HCW (96.9% vs. 87.8%; *p* < 0.016). Ct-values for the RT-PCR results were available for 332 (66.4%) individuals. The majority of Ct-values are within the range of 31–40 (67.9%) with no difference between HCW and non-HCW. Additional diagnoses were more common among non-HCW than among HCW: pneumonia (49.3% vs. 16%; *p* < 0.001) and acute respiratory distress syndrome (ARDS) (13.3% vs. 3%; p = 0.001). Among the 500 COVID-19-confirmed cases, 61 (12.2%) died. Mortality was higher among non-HCW compared with HCW (16.3% vs. 0.7%; *p* < 0.001).

**Table 1:** Epidemiologic and clinical characteristics of healthcare and non-healthcare workers COVID-19 inpatients in San Lazaro Hospital, January 2020 to October 2020

Characteristics	Total (*n* = 500)	HCW cases (*n* = 133)	non-HCW (*n* = 367)	*p*-value
Age (years)				
Mean (SD)	48 (17)	41 (11.5)	51 (18)	< 0.001
Median (IQR)	48 (34–61)	40 (31–50)	53 (38–64)	
Age group (years)				
0–10 years old	7 (1.4)	–	7 (1.9)	< 0.001
11–20 years old	9 (1.8)	–	9 (2.4)	
21–40 years old	158 (31.6)	67 (50.4)	91 (24.8)	
41–60 years old	195 (39.0)	58 (43.6)	137 (37.3)	
61–80 years old	115 (23.0)	8 (6.0)	107 (29.2)	
80+ years old	16 (3.2)	–	16 (4.4)	
Sex				
Female	221 (44.2)	63 (47.4)	158 (43.0)	0.390
Male	279 (55.8)	70 (52.6)	209 (57.0)	
Nationality				
Filipino	498 (99.6)	133 (100.0)	365 (99.5)	1.000
Chinese	2 (0.4)	–	2 (0.5)	
Exposure history				
International travel history within 14 days prior to admission	9 (1.8)	0	9 (2.4)	< 0.001
Exposed to a confirmed case	21 (4.2)	10 (7.5)	11 (3.0)	
Risk of exposure in workplace	105 (21.0)	96 (72.2)	9 (2.4)	
No identified exposure	365 (73.0)	27 (20.3)	339 (92.1)	
Reported symptoms				
Fever	281 (56.2)	54 (40.6)	227 (61.8)	< 0.001
Cough	381 (76.2)	80 (60.1)	301 (82.0)	< 0.001
Colds	142 (28.4)	51 (38.3)	91 (24.8)	0.003
Difficulty of breathing	185 (37.0)	18 (13.5)	167 (45.5)	< 0.001
Headache	22 (4.4)	4 (3.0)	18 (4.9)	0.464
Malaise/fatigue	45 (9.0)	6 (4.5)	39 (10.6)	0.034
Diarrhoea	34 (6.8)	5 (3.8)	29 (7.9)	0.112
Anosmia	37 (7.4)	6 (4.5)	31 (8.4)	0.176
Ageusia	33 (6.6)	6 (4.5)	27 (7.4)	0.312
Loss of appetite	23 (4.6)	0	23 (6.3)	0.001
Co-morbidities				
Hypertension	177 (35.4)	37 (27.8)	140 (38.1)	0.033
Diabetes	87 (17.4)	13 (9.8)	74 (20.2)	0.007
Bronchial asthma	22 (4.4)	7 (5.3)	15 (4.1)	0.622
HIV	6 (1.3)	0	6 (1.6)	0.349
Cardiac disease	20 (4.0)	3 (2.3)	17 (4.6)	0.306
Tuberculosis (any form)	41 (8.2)	4 (3.0)	37 (10.1)	0.009
At least one underlying disease	258 (51.6)	53 (40.0)	205 (55.9)	0.002
Clinical status				
Asymptomatic	0	0	0	< 0.001
Mild	136 (27.2)	63 (47.4)	73 (19.9)	
Moderate	162 (32.4)	56 (42.1)	106 (28.9)	
Severe	121 (24.2)	7 (5.3)	113 (31.1)	
Critical	70 (14.0)	7 (5.3)	63 (17.2)	
Intubated	29 (5.8)	0	29 (100.0)	< 0.001
Duration between the onset of symptoms and admission				
Mean (SD)	7 (13)	6 (3.5)	8 (15)	< 0.001
Median (IQR)	6 (4–9)	5.5 (4–8)	7 (5–10)	
0–14 days	448 (89.6)	126 (96.9)	322 (87.8)	0.016
15–30 days	39 (7.8)	4 (3.1)	35 (9.5)	
> 30 days	6 (1.2)	–	6 (1.6)	
Hospitalisation days				
Mean (SD)	12 (8)	11 (5)	12 (9)	0.340
Median (IQR)	10 (7–14)	10 (7–12)	10 (7–15)	
Other diagnoses				
Pneumonia	202 (40.4)	21 (16.0)	181 (49.3)	< 0.001
Upper respiratory tract infection	24 (4.8)	9 (6.8)	15 (4.1)	0.238
Acute gastroenteritis	13 (2.6)	3 (2.3)	10 (2.7)	1.000
Acute respiratory distress syndrome	52 (10.4)	4 (3.0)	48 (13.1)	0.001
Ct-value distribution		n = 105	N = 227	0.317
11–20	5 (1.5)	2 (1.9)	3 (1.3)	
21–30	98 (30.0)	37 (35.2)	63 (27.7)	
31–40	222 (67.9)	66 (62.9)	159 (70.0)	
> 40	2 (0.6)	–	2 (0.9)	
Outcome				
Died	61 (12.2)	1 (0.7)	60 (16.3)	< 0.001
Discharged	439 (87.8)	132 (99.3)	307 (83.6)	

Table 2 shows the characteristics of the 367 non-HCW by mortality. No death occurred for the 0–10 years age group, but deaths were recorded across all other age groups. Compared to those who recovered, individuals who died were more likely to be older (*p* < 0.001), male (p = 0.015), report difficulty of breathing (*p* < 0.001), be HIV positive (*p* = 0.008), be intubated (*p* < 0.001), categorised as severe or critical (*p* < 0.001), have a shorter mean hospital stay (*p* < 0.001), or have an alternative diagnosis of pneumonia (*p* < 0.001) or ARDS (*p* < 0.001). There were no significant associations in mortality by nationality, exposure history, duration between onset of symptoms and admission, and Ct-value.

**Table 2:** Associations with mortality among 367 non-HCW COVID-19 inpatients in San Lazaro Hospital, January to October 2020

Characteristics	All cases (*n* = 367)	Died (*n* = 60)	Discharged (*n* = 307)	*P*-value
Age (years)				
Mean (SD)	51 (18)	59 (20)	49 (17)	< 0.001
Median (IQR)	53 (38–64)	65 (41–75)	51 (37–62)	
Age group (years)				
0–10 years old	7 (1.9)	0	7 (100.0)	< 0.001
11–20 years old	9 (2.4)	2 (22.2)	7 (77.8)	
21–40 years old	91 (24.8)	11 (12.1)	80 (87.9)	
41–60 years old	137 (37.3)	14 (10.2)	123 (89.8)	
61–80 years old	107 (29.2)	24 (22.4)	83 (77.6)	
80+ years old	16 (4.4)	9 (56.2)	7 (43.8)	
Sex				
Female	158 (43.0)	17 (10.8)	141 (89.2)	0.015
Male	209 (57.0)	43 (20.6)	166 (79.4)	
Nationality				
Filipino	365 (99.5)	59 (16.6)	306 (83.8)	0.197
Chinese	2 (0.5)	1 (50.0)	1 (50.0)	
Exposure history				
International travel history within 14 days prior to admission	9 (2.5)	2 (22.2)	7 (77.8)	0.247
Exposed to a confirmed case	11 (3.0)	0	11 (100.0)	
Risk of exposure in workplace	9 (2.5)	0	9 (100.0)	
No identified exposure	338 (92.0)	58 (17.2)	280 (82.8)	
Reported symptoms				
Fever	227 (61.8)	39 (17.2)	188 (82.8)	0.583
Cough	301 (82.0)	53 (17.6)	248 (82.4)	0.200
Colds	91 (84.8)	9 (9.9)	82 (90.1)	0.071
Difficulty of breathing	167 (45.5)	48 (28.7)	119 (71.3)	< 0.001
Headache	18 (4.9)	2 (11.1)	16 (88.9)	0.749
Malaise/fatigue	39 (10.6)	3 (7.7)	36 (92.3)	0.168
Diarrhoea	29 (7.9)	1 (3.5)	28 (96.5)	0.050
Anosmia	31 (8.4)	1 (3.2)	30 (96.7)	0.041
Ageusia	27 (7.4)	1 (3.7)	26 (96.3)	0.065
Loss of appetite	23 (6.3)	5 (21.7)	18 (78.3)	0.558
Co-morbidities				
Hypertension	140 (38.1)	22 (15.7)	118 (84.3)	0.796
Diabetes	74 (20.2)	14 (18.9)	60 (81.1)	0.486
Bronchial asthma	15 (4.1)	0	15 (100.0)	0.145
HIV	6 (1.6)	4 (66.7)	2 (33.3)	0.008
Cardiac disease	17 (4.6)	3 (17.6)	14 (82.3)	0.747
Tuberculosis (any form)	37 (10.1)	10 (27.0)	27 (73.0)	0.097
At least one underlying disease	205 (55.8)	36 (17.6)	169 (82.4)	0.480
Clinical status				
Asymptomatic	0	0	0	< 0.001
Mild	73 (19.9)	1 (1.4)	71 (98.6)	
Moderate	106 (28.9)	0	104 (98.1)	
Severe	114 (31.1)	23 (20.2)	89 (77.4)	
Critical	63 (17.2)	36 (48.7)	38 (51.3)	
Intubated	29 (7.9)	28 (96.5)	1 (3.5)	< 0.001
Duration between the onset of symptoms and admission				
Mean (SD)	8 (15)	5 (33)	8 (7)	0.229
Median (IQR)	7 (5–10)	6 (4–9)	7 (5–10)	
0–14 days	322 (88.7)	53 (16.4)	269 (87.6)	0.047
15–30 days	35 (9.6)	4 (11.4)	31 (88.6)	
> 30 days	6 (1.7)	3 (50.0)	3 (50.0)	
Hospitalisation days				
Mean (SD)	12 (9)	7 (9)	13 (9)	< 0.001
Median (IQR)	10 (7–15)	5 (1–10)	11 (8–16)	
Other diagnoses				
Pneumonia	181 (49.3)	49 (27.1)	132 (72.9)	<0.001
Upper respiratory tract infection	15 (4.1)	0	15 (100.0)	0.080
Acute gastroenteritis	10 (2.7)	0	10 (100.0)	0.378
Acute respiratory distress syndrome	48 (13.1)	33 (68.7)	15 (31.2)	<0.001
Ct-values distribution	N = 227	N = 36	N = 191	
11–20	3 (1.3)	1 (33.3)	2 (66.70)	0.178
21–30	63 (27.7)	14 (22.2)	49 (77.8)	
31–40	159 (70.0)	20 (12.6)	139 (87.4)	
> 40	2 (0.9)	1 (50.0)	1 (50.0)	

## Conclusions

In this study, we describe the epidemiological and clinical characteristics of the first 500 confirmed COVID-19 individuals admitted to an infectious disease referral hospital in Metro Manila. There were significant differences in characteristics, symptomatology, and outcomes between healthcare and non-healthcare workers. This likely reflects the changes in policy for admission and access to testing, with many frontline healthcare workers with mild symptoms admitted to the hospital in the early days of the epidemic [13]. Non-healthcare workers were more likely to report cough, fever, and difficulty of breathing and have pneumonia and more severe disease [14, 15].

The mortality rate among non-HCW was 16.4%, comparable to the 17.5% mortality reported among the first 200 COVID-19 cases at the Philippine General Hospital and a similar population in Indonesia with a mortality rate of 12% [15, 16], but lower compared to those reported in large cohorts in high-income countries [17, 18]. Older age was associated with mortality as consistently reported elsewhere [14, 17, 18]. The presence of an underlying illness among COVID-19 non-HCW (82.4%) is similar to other case series reported in North America (88%) [17] and the UK (77.5%) [18], but comorbidities of diabetes and hypertension were not associated with mortality in our study as would be expected [14, 19]. This may reflect reporting biases or our limited sample size. HIV positivity was significantly associated with mortality (p = 0.008). As the tertiary referral hospital for infectious diseases in the country, SLH preferentially caters to complex cases of infectious diseases even before the pandemic started, such as HIV. Further investigation suggested that these individuals included referrals from other hospitals with medical problems in addition to COVID-19.

Our analysis has some limitations. The retrospective design of our study and reliance on the available data from the CIF meant that some variables were incomplete. Details on the patients’ course in the wards and treatment received were also not available. As data from an infectious disease referral hospital, caution should be considered in interpreting the results in the context of the general population. In conclusion, we report various sociodemographic and clinical characteristics associated with increased COVID-19 mortality among hospitalised individuals in Metro Manila, Philippines. Our results support the country’s national guideline on COVID-19 vaccination which prioritises healthcare workers, the elderly population, and people with comorbidities and immunodeficiency states.

## Data Availability

The dataset for this study is available from the corresponding author and San Lazaro Hospital on a reasonable request. Data without names and identifiers will be made available after approval from the corresponding author and San Lazaro Hospital.

## Abbreviations

ARDS: Acute respiratory distress syndrome
CIF: Case investigation form
Ct-value: Cycle threshold value
DOH: Department of Health
HCW: Healthcare workers
NCR: National Capital Region
non-HCW: Non-healthcare worker
SLH: San Lazaro Hospital
SLH – ED: San Lazaro Hospital – Epidemiology Department
